# More foreign-accented but more comprehensible: Attrition and amelioration of L1 speech in proficient L2 learners

**DOI:** 10.12688/f1000research.148193.1

**Published:** 2024-08-01

**Authors:** Kakeru Yazawa, Takayuki Konishi, Rubén Pérez-Ramón, Mariko Kondo

**Affiliations:** 1 Institutes of Humanities and Social Sciences, University of Tsukuba, Tsukuba, Ibaraki, 305-8571, Japan; 2 School of Languages and Communication, Kobe University, Hyogo, Kobe, 657-8501, Japan; 3 School of International Liberal Studies, Waseda University, Shinjuku, Tokyo, 169-8050, Japan; 4 Gradual School of International Culture and Communication Studies, Waseda University, Shinjuku, Tokyo, 169-8050, Japan

**Keywords:** Foreign accent, comprehensibility, proficiency, length of residence, phonetic attrition, J-AESOP corpus, category compactness, vowel dispersion

## Abstract

**Background:**

There is an increasing interest in cross-linguistic influences of the second language (L2) on the first (L1), but its communicative impact remains to be elucidated. This study investigates how L2 learners’ L1 pronunciation is perceived as foreign-accented and (in) comprehensible as a function of their L2 learning experience and proficiency levels.

**Methods:**

Read speech of 154 L1 Japanese learners of L2 English in the J-AESOP corpus was examined, where approximately one-third of them had lived in English-speaking countries and the rest had never lived outside of Japan. Their L1 speech was rated by another group of native Japanese listeners for accentedness and comprehensibility (from October 25, 2022 to August 20, 2023), while their L2 speech was previously rated by native American English listeners for nativelikeness or proficiency. The speakers’ vowel acoustics were also examined.

**Results:**

More proficient L2 speakers were perceived as more foreign-accented in their L1, but only if they had lived overseas; their length of residence abroad predicted the degree of perceived accentedness. In contrast, more proficient L2 speakers were consistently perceived as more comprehensible in the L1, regardless of prior overseas experience. Acoustic analyses indicated that perceived accentedness is associated with a clockwise chain shift of all vowel categories in the vowel space. It was also revealed that the dispersion, rather than compactness, of vowel production contributed to perceived comprehensibility, although the degree of L1 vowel dispersion did not predict L2 proficiency.

**Conclusions:**

The overall results suggest two main conclusions. First, perceptible L1 foreign accent likely results from L1 disuse rather than L2 interference, thereby L1 pronunciation differs from native norms at a system-wide rather than category-specific level. Second, L2 learning has a positive influence on perceived L1 comprehensibility, rather than individuals with clearer and more comprehensible L1 speech being inherently better L2 learners.

## 1. Introduction

“Foreign accent” is a term commonly used to refer to speech characteristics that differ from what native speakers recognize as a native accent. It is usually used in a situation where a speaker’s first language (L1) affects their second language (L2), a phenomenon known as “forward transfer” (or simply “transfer”), which has been widely studied in the field of L2 speech research (e.g., Kondo and Pérez-Ramón (2023) and Yazawa et al. (2023a) on Japanese-accented English). However, foreign accents can also emerge in the ‘opposite’ situation, that is, when a speaker’s L2 learning experience affects their L1 pronunciation ( de Leeuw et al., 2010; Kornder, 2022; Laméris et al., 2024). This phenomenon, sometimes called “backward transfer” ( Cook, 2003), has received increasing attention in recent years, although much remains to be elucidated about its nature. The purpose of the current study is to address some of the less documented issues in phonetic backward transfer, including its impact on perceived L1 accentedness and comprehensibility, in relation to the speaker’s L2 learning experience and proficiency levels.

Given the recent surge of interest in phonetic backward transfer, with various terms used to describe its specific aspects, it is important to clarify the terminology that we will use in this study. One of the most commonly used terms for L2-induced L1 pronunciation change is “phonetic drift” ( Chang, 2019). While the term itself was first introduced by Sapir (1921) to refer to diachronic sound change in ‘macro’ language (i.e., language at the level of the speech community, such as English) and is still used in that sense, our use of the term follows that of Chang (2019) in that we refer to sound change over time within ‘micro’ language (i.e., language at the level of the individual, namely an idiolect). The precise definition we adopt from Chang (2019) is “ *L2-influenced phonetic change in an individual’s L1 system*” (p. 191) that is “short-term” (p. 192) and “attributable to *recent* L2 experience” (p. 192). When this drift becomes chronic due to cumulative use of the L2, we use the term “phonetic attrition” ( de Leeuw, 2019). Attrition can, in an extreme case, cause a bilingual’s L1 speech to be perceived as foreign-accented by monolingual listeners of the language, although this does not necessarily imply that short-term phonetic drift never affects the perceived accentedness of L1 speech. To encompass both cases of drift and attrition, we will also employ a more general (but lesser-used) umbrella term, “phonetic change,” in this paper.

The growing body of literature on L1 phonetic change has been thoroughly reviewed by Kartushina et al. (2016). The paper identifies several factors that seem to affect the likelihood and magnitude of phonetic change, such as the age of onset (AoO) of L2 acquisition, the level of L2 proficiency, and the amount of L1 use, among others. The reviewed findings can be summarized as follows: whereas simultaneous bilinguals are capable of attaining and maintaining nativelike competence in both languages, sequential bilinguals are more prone to L1 phonetic drift and attrition; even novice L2 learners can show L1 drift due to ‘novelty’ effects, which is somewhat diminished for intermediate learners, whereas highly proficient L2 speakers show signs of attrition; less frequent L1 use, which is often concordant with L2 dominance and proficiency, results in greater L1 phonetic change. However, one of the main challenges in this type of setting, where the speaker is fully immersed in an L2-speaking—and thus non-L1-speaking—environment, is that the above factors are difficult to disentangle from each other. For example, the effect of L2 proficiency is intertwined with that of L1 disuse, as they often go hand in hand (especially in migrant settings). This is one of the key issues that we aim to unravel in the current study.

A fundamental assumption underlying many of the previous studies on L1 phonetic change is that the phenomenon is driven by the developing and newly established sound categories in the learner’s L2 system. This assumption derives largely from the (revised) Speech Learning Model (SLM(-r); Flege, 1995; Flege & Bohn, 2021), which proposes that L1 and L2 phonetic categories exist in a common space and constantly influence each other in a bidirectional manner. Thus, studies have reported cases where an L1 segmental category assimilates (i.e., moves closer) to or dissimilates (i.e., moves away) from its closest L2 category, with the AoO being a potential factor affecting which process takes place (see Kartushina et al. (2016) for details). However, other studies have suggested an alternative possibility that L1 phonetic change occurs at a broader, system-wide level. Guion (2003) investigated L1 Quechua speakers who differed in their L2 Spanish AoO and found that L1 Quechua vowels of early bilinguals (who acquired a five-vowel system for the L2) generally had lower first formant (F1) values than those of late bilinguals (who maintained a three-vowel, L1 Quechua-like system). This suggests that learning new L2 Spanish vowel categories shifted the L1 Quechua vowel space upward, which cannot be explained by segment-by-segment assimilation (or dissimilation) between the two languages since not all Spanish vowels are higher (or lower) than in Quechua. The author proposed that this system-wide raising of L1 vowels serves to enhance their perceptual distinctiveness from the L2 vowels. Mayr et al. (2012) and Turner (2023) reported similar cases but in the opposite direction, where increased L2 exposure caused a systematic increase in F1 values (i.e., downward shift) of all L1 vowels, in Dutch-English bilinguals and L1 English learners of L2 French, respectively. An important finding of Turner’s (2023) longitudinal study is that learners showed a partial reversal of phonetic drift after re-immersion into the L1-speaking environment, which again occurred at a system-wide rather than category-specific level. Interestingly, none of the above three studies observed a change in second formant (F2) frequencies.

The majority of previous research has focused on how L1 phonetic change occurs at the acoustic level, including F1 and F2 frequencies of vowels, voice onset time (VOT) of stops, center of gravity of fricatives, to name a few. The communicative impact of such acoustic changes, however, has been relatively understudied. De Leeuw et al. (2010) found that L1 German speakers who had moved to Canada or to the Netherlands at an average age of 27 and had lived there for an average of 37 years were perceived as having a noticeable foreign accent, with some even being recognized as non-native speakers. A longitudinal study by Kornder (2022) also found that Arnold Schwarzenegger, who was born in Austria in 1947 and moved to the United States in 1968, has a perceptible foreign accent in his recent L1 German (2010-2017) that was absent in the early stage of his career (1977-1989). More relevant to the current study, Laméris et al. (2024) examined accentedness ratings of Japanese-English bilingual returnees (Japanese children who returned to Japan after living in an English-speaking environment for several years) at three time points (upon return to Japan, one year after return, and five years after return) and found a decrease in L1 foreign accent as early as one year after return; the overall degree of L1 accentedness was more pronounced for returnees with earlier English AoO and increased L2 exposure while abroad. Taken together, these studies suggest that L2 learning experience can affect the perceived ‘authenticity’ of L1 speech, which seems to be reversible to some extent after re-immersion into the L1 environment. However, it remains unclear which acoustic characteristics contribute to the degree of perceived foreign accentedness and how. Also, the effects of L1 phonetic change on perceived comprehensibility (i.e., how easy an utterance is to understand) has rarely been studied. The only study we are aware of that addressed the above two points is Kornder (2022), who found a change in stop VOT and vowel formants but no significant difference in perceived comprehensibility between early and late L1 productions of Arnold Schwarzenegger. However, due to the nature of the study focusing on one speaker, it is not known how generalizable the results are.

Based on the literature reviewed above, the current study aims to address the following research questions to shed more light on the communicative impact of L1 phonetic change. Our first question concerns the perceived accentedness of L1 speech: what is the source of L1 foreign accent, L2 interference or L1 disuse? Since all of the previous studies on L1 foreign accent reviewed above ( de Leeuw et al., 2010; Kornder, 2022; Laméris et al., 2024) investigated a migrant population that had been using their L2 as their dominant language for many years, it is unclear whether their L1 accent is caused by an interference from the L2 system or by a long-term disuse of the L1 system. Our second question is related to the first: does L1 foreign accent reflect a category-specific process or a system-wide shift? Regardless of the source of L1 accent, the L1 system of foreign-accented speakers should exhibit some acoustic characteristics that are absent in or different from non- and less-accented speakers, which may take the form of individual L1 sound categories assimilating or dissimilating to L2 categories, as is usually assumed, or of all L1 categories changing at a systematic level, as some studies have suggested ( Guion, 2003; Mayr et al., 2012; Turner, 2023). Finally, our third question concerns the comprehensibility of L1 speech: what is the influence of L2 learning on L1 comprehensibility? In the case of forward transfer, it is known that a strong degree of accentedness does not necessarily imply a loss of comprehensibility or intelligibility ( Munro & Derwing, 1995). However, the same pattern may not necessarily hold for backward transfer because, according to Cook (2003), the effects of the L2 on the L1 can be positive (e.g., enhanced metalinguistic skills), negative (e.g., loss of L1 competence), or neutral (not ‘better’ or ‘worse’ but simply ‘different’).

To answer these questions, we conduct a large-scale rating study of perceived accentedness and comprehensibility in L1 Japanese speech of L2 English learners with diverse learning experience and proficiency levels, using the J-AESOP corpus ( Kondo et al., 2015). The current study builds on our previous pilot investigation using the same corpus ( Yazawa et al., 2023b), which suggested a potential influence of L2 proficiency on L1 accentedness and comprehensibility, but this finding needs to be confirmed with more raters than was tested ( *n* = 10). Our previous study also did not probe into the speakers’ L2 learning experience (e.g., whether they had lived in an L2-speaking environment and for how long) and the acoustic characteristics of the speech, which we aim to complement in the current investigation. In what follows, we first present the rating results in Section 2, followed by the acoustic analysis in Section 3. Section 4 discusses the implications of the results, limitations of the current study, and directions for further research.

## 2. Perceptual rating

### 2.1 Speakers

The J-AESOP corpus contains speech data of 183 L1 Japanese learners of L2 English as well as 20 L1 English learners of L2 Japanese. The current study focuses on 154 L1 Japanese speakers (94 female, 60 male) who had never lived overseas in a non-English-speaking country (e.g., Spain) in order to control for the potential influence of a third language (L3). The speakers were all undergraduate or graduate students at universities in and around Tokyo, Japan, between the ages of 18 and 38 (mean = 20.04, standard deviation = 2.10). Approximately two-thirds of them ( *n* = 99; 50 female, 49 male) had spent their entire lives in Japan, with most of them having studied English for six years (age 13-18) as part of their compulsory education in secondary schools. They had also received some English instruction in college, the quality and quantity of which varied depending on the courses in which they were enrolled. The remaining third ( *n* = 55; 44 female, 11 male) had some experience of living in an English-speaking country (32 in the US, 10 in the UK, nine in Australia, eight in Canada, three in New Zealand, with some having lived in more than one country), with the length of residence (LOR) ranging from one month to 11 years (mean = 27.45 months, standard deviation = 32.46 months), but none of them were considered simultaneous bilinguals. Their language learning background while in Japan was similar to that described above, except that some speakers attended international schools as part of their secondary education.

### 2.2 Materials

The speech materials to be rated are taken from Tasks 6_01 and 6_02 of the J-AESOP corpus, where the speakers read aloud the English and Japanese versions of “The North Wind and the Sun” ( International Phonetic Association, 1999) shown in Table 1 and Table 2, respectively. The trisecting of the text in Table 1 is for rating purposes only (see Section 2.4.1), and the speakers read the passage as a whole.

**Table 1.** Full text of the English version of “The North Wind and the Sun” (Task 6_01).

Passage
*The North Wind and the Sun were disputing which was the stronger, when a traveler came along wrapped in a warm cloak. They agreed that the one who first succeeded in making the traveler take his cloak off should be considered stronger than the other.*
*Then the North Wind blew as hard as he could, but the more he blew the more closely did the traveler fold his cloak around him; and at last the North Wind gave up the attempt.*
*Then the Sun shone out warmly, and immediately the traveler took off his cloak. And so the North Wind was obliged to confess that the Sun was the stronger of the two.*

**Table 2.** Full text of the Japanese version of “The North Wind and the Sun” (Task 6_02).

Passage
*Arutoki Kitakaze to Taiyou ga chikarakurabe o shimashita. Tabibito no gaitou o nugaseta hou ga kachi to iu koto ni kimete, mazu Kitakaze kara hajimemashita. Kitakaze wa, “Nani, hitomakuri ni shite miseyou,” to, hageshiku fukitatemashita. Suruto tabibito wa, Kitakaze ga fukeba fukuhodo gaitou o shiQkarito karada ni kuQtsukemashita. KoNdo wa Taiyou no baN ni narimashita. Taiyou wa kumo no aida kara yasahii kao o dashite, atatakana hikari o okurimashita. Tabibito wa daNdaN yoi kokoromochi ni natte, shimai ni wa gaitou o nugimashita. Sokode kitakaze no make ni narimashita.*

### 2.3 Raters

**2.3.1 English raters**

Four phonetically trained native American English listeners were recruited to rate the audio samples of Task 6_01 as part of a previous research project ( Konishi, 2022). All of them had a graduate degree in phonetics or a related field and were familiar with the L2 English pronunciation of L1 Japanese speakers. Such experienced raters are known to be able to consistently rate various aspects of speech, including segmental accuracy, word stress, intonation, and rhythm ( Saito et al., 2017).

**2.3.2 Japanese raters**

Twenty-six native Japanese listeners (22 female, 4 male) were recruited to rate the audio samples of Task 6_02 for the current study (this sample includes the 10 listeners in Yazawa et al. (2023b)). They were all undergraduate or graduate students at the University of Tsukuba, Ibaraki, Japan, between the ages of 19 and 25 (mean = 21.04, standard deviation = 1.93). Twenty of them had never lived outside of Japan, while the remaining six had studied abroad in an English-speaking country (three in the UK, two in the US, and one in Australia) for one to ten months. Although the raters were not as linguistically experienced as the four native American English raters, it has been shown that such listeners can coherently evaluate global accentedness and comprehensibility ( Saito et al., 2017).

### 2.4. Procedure

**2.4.1 English rating**

The native American English listeners were provided with all the trisected audio samples of Task 6_01 for self-paced rating. They were instructed to listen to each trisection and rate the speech according to four criteria (segmental accuracy, prosody, fluency, and nativelikeness) on a 10-point scale each. The current study focuses on the nativelikeness score, where a value of 1 corresponds to “strongly foreign-accented” and a value of 10 corresponds to “free of foreign accent.” Once the rating was completed, the obtained scores were averaged across the trisections per speaker, yielding a single nativelikeness score for each speaker as assessed by one rater. The trisection scheme was to improve the accuracy of the rating by having a rater evaluate the same speaker three times, which also made the resulting scores non-integer (i.e., 1.00, 1.33, 1.67, etc.). Raters received monetary compensation for their time and effort. More details of the English rating can be found in Konishi (2022).

**2.4.2 Japanese rating**

The native Japanese listeners rated the audio samples of Task 6_02 online via Gorilla Experiment Builder ( Anwyl-Irvine et al., 2020). They signed a written consent form and completed a language background questionnaire prior to participation. After a brief tutorial and a practice session, the raters listened to a speaker’s audio sample presented in random order. The use of headphones or earphones was encouraged, but not required, since the use of headphones is unlikely to have any substantial effect on the results of most online perception experiments ( Sanker, 2023). The raters then indicated the perceived impression of global accentedness and comprehensibility using two horizontal sliders. The slider for accentedness read “strong foreign accent” on one end and “no foreign accent” on the other, and the slider for comprehensibility read “difficult to understand” on one end and “easy to understand” on the other, in Japanese. The slider values were internally coded from 0 to 100 with integer increments, with a larger value corresponding to a higher level of perceived accentedness or comprehensibility. A pause screen was displayed after each trial to allow the raters to take a short break at any time during the rating. All raters completed their ratings within one week and received monetary compensation for their time and effort. The language background of the speakers was disclosed only after they completed the task.

The above procedural design, including the consent form and the questionnaire, was reviewed and approved by the Research Ethics Committee of the Institutes of Humanities and Social Sciences, University of Tsukuba on May 17, 2022 (approval number 2022-3). All procedures were conducted in accordance with the ethical standards of the Helsinki Declaration.

### 2.5 Results and analysis

**2.5.1 English scores**

**Figure 1.**
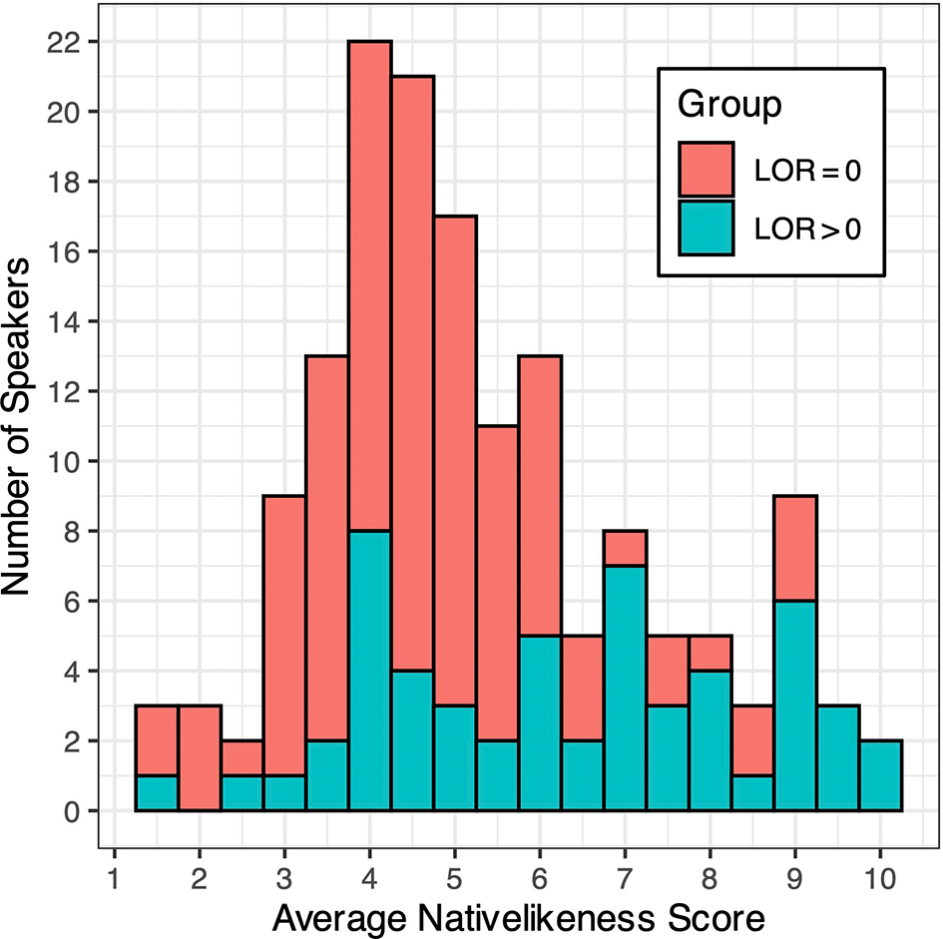
Mean English nativelikeness scores of all speakers, conditioned by their length of residence (LOR) abroad.

Figure 1 shows the mean L2 English nativelikeness scores of all 154 L1 Japanese speakers (averaged across four native American English raters), conditioned by their presence and absence of previous residence in an English-speaking country. The intraclass correlation coefficient (ICC) using a two-way random-effects model for consistency ( Koo & Li, 2016) was 0.892, indicating a very high level of inter-rater consistency. It can be seen that the scores are fairly evenly distributed, ranging from nearly 1 (“strongly foreign-accented”) to 10 (“free of foreign accent”). While speakers with overseas experience (i.e., LOR > 0) tend to have higher scores than those without (i.e., LOR = 0), there is a significant overlap in L2 nativelikeness score between the two groups (i.e., LOR > 0 vs. LOR = 0). Therefore, by-group investigation of the data would help us to disentangle the potential effect of L2 proficiency (as represented by nativelikeness score) from that of L1 disuse associated with overseas experience (as represented by LOR).

**2.5.2 Japanese scores**

**Figure 2.**
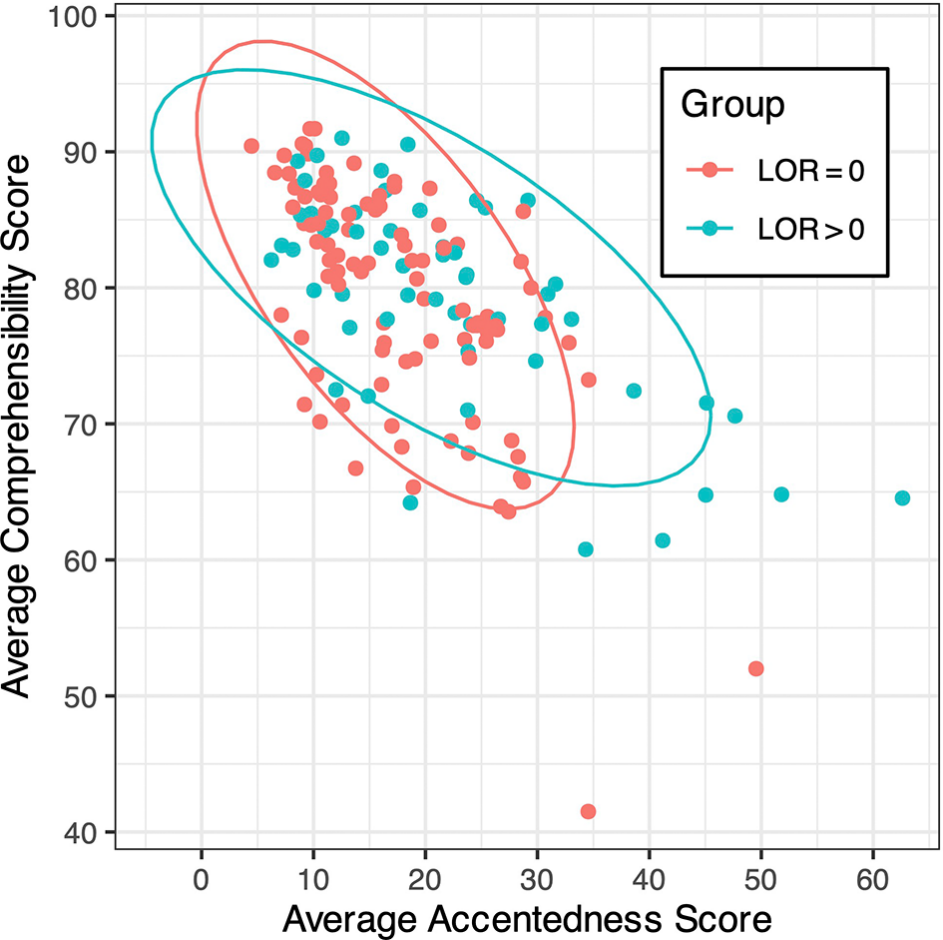
Mean Japanese accentedness and comprehensibility scores of all speakers, conditioned by their LOR abroad.

Figure 2 shows the mean L1 Japanese accentedness and comprehensibility scores of the same 154 speakers (averaged across 26 native Japanese raters), conditioned by their previous experience of overseas residence. The ICC was 0.279 for accentedness and 0.271 for comprehensibility, indicating a weak but acceptable level of inter-rater consistency ( Fleiss & Cohen, 1973). The confidence ellipses for L1 accentedness scores indicate that most speakers were not perceived as very accented, although some speakers, those with overseas experience (i.e., LOR > 0) in particular, were judged to be moderately accented. The ellipses for L1 comprehensibility scores indicate that most speakers were perceived as generally comprehensible, with a few speakers without overseas experience (i.e., LOR = 0) being apparent outliers. The two types of scores are negatively correlated (Pearson’s *r* = -0.62), suggesting that more accented speech tended to be perceived as less comprehensible. However, it is still possible that these score types are differentially related to L2 experience and proficiency, as will be illustrated below.

**2.5.3 Relationship between English and Japanese scores**

**Figure 3.**
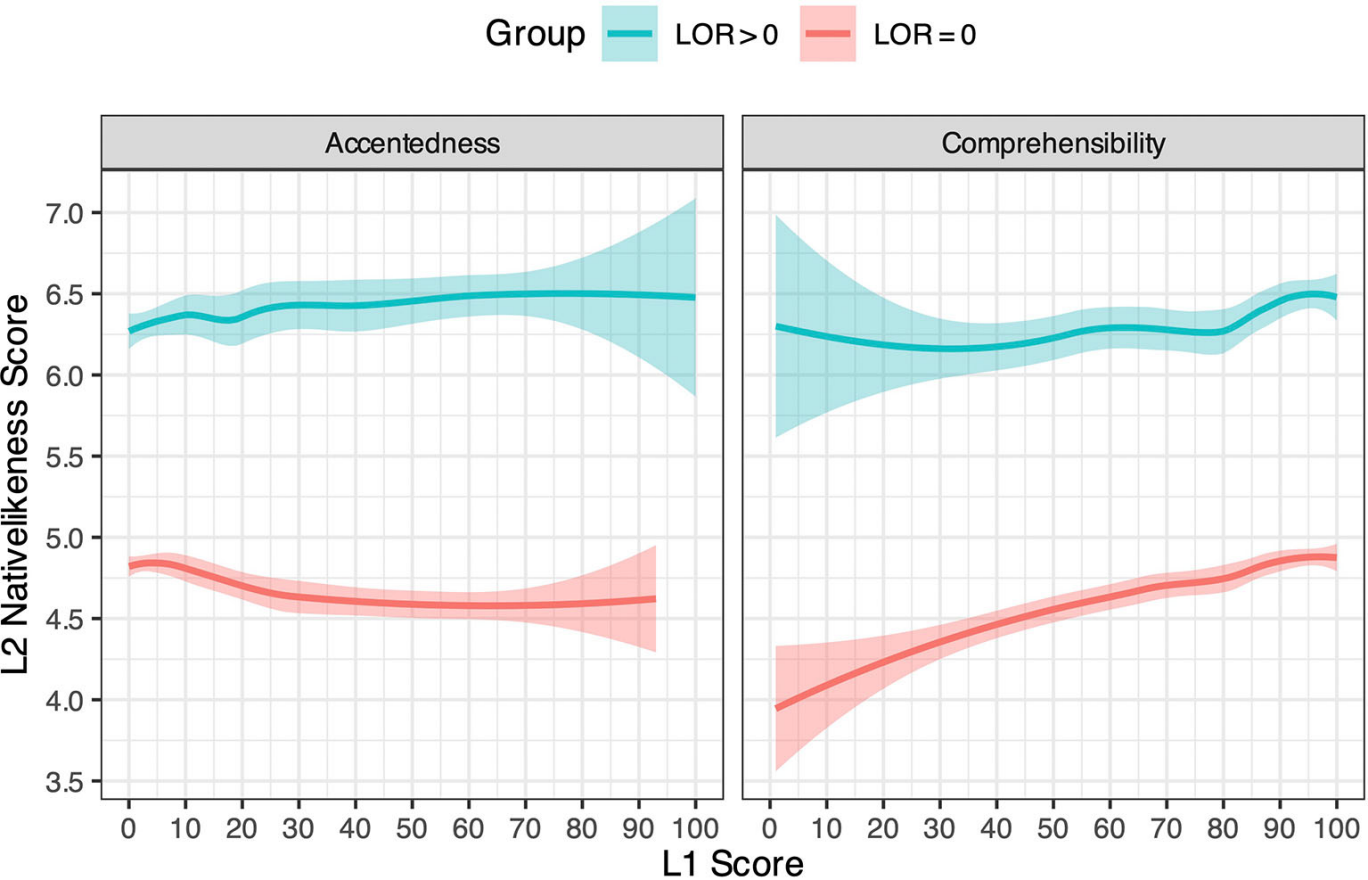
Smoothed means of L1 Japanese and L2 English scores, conditioned by speakers’ LOR abroad.

Figure 3 shows the relationship between the L2 English and L1 Japanese scores, again conditioned by the speakers’ overseas experience. Regarding the L1 accentedness score (left side of the figure), distinct patterns can be identified between the two LOR groups. For speakers with LOR > 0, a higher L1 accentedness score seems to be associated with a slightly higher L2 nativelikeness score, suggesting that more proficient L2 speakers tended to be perceived as more foreign-accented in their L1 speech. Given that there was a positive correlation between LOR (in months) and L2 nativelikeness score for these speakers (Pearson’s *r* = 0.59), this may also suggest that the longer they had stayed in an English-speaking country, the more accented their Japanese was judged to be. This explanation is further supported by the opposite pattern observed for speakers with LOR = 0, where a higher L1 accentedness score seems to be associated with a lower L2 nativelikeness score. Taken together, these results suggest that the experience of living overseas, rather than L2 proficiency per se, contributes to perceived L1 accentedness. In contrast, regarding L1 comprehensibility (right side of the figure), both LOR groups show the same tendency where a higher L1 comprehensibility score leads to a higher L2 nativelikeness score. This suggests that more proficient L2 speakers were generally perceived as more comprehensible in their L1 speech, regardless of prior experience abroad.

To test whether the above observations hold at statistically significant levels, a cumulative link mixed model (CLMM) was fitted to the response data per speaker group (i.e., LOR = 0 or LOR > 0), using the *ordinal* package ( Christensen, 2023) in R ( R Core Team, 2024). The structure of the model was as follows:

clmm(L2.nativelikeness∼L1.accentedness+L1.comprehensibility+(1|EN.rater)+(1|JP.rater),df.subset) (1)

where the dependent variable of L2 nativelikeness score was predicted by the fixed effects of L1 accentedness and comprehensibility scores, with random intercepts for native American English and native Japanese raters. Random slopes were not included because the models did not converge. Table 3 and Table 4 show the results of the two models. For speakers with LOR > 0 ( Table 3), both fixed effects were statistically significant, and the positive estimates confirm that more proficient L2 speakers tended to be perceived as more accented and more comprehensible in the L1. In contrast, only the effect of L1 comprehensibility was significant for speakers with LOR = 0 ( Table 4), and thus L2 proficiency does not reliably predict L1 accentedness for these speakers. One caveat, however, is that the estimates of the statistically significant effects are not very large. For example, the estimate for L1 accentedness in Table 3 is about 0.007, and thus an increase in L1 accentedness score from 0 to 100 would result in an increase in L2 nativelikeness score of 0.7 on a 10-point scale, for speakers with LOR > 0 (see Figure 3).

**Table 3.** Result of model (1) for speakers with LOR > 0.

	Estimate	Std. Error	*Z*	*p*
L1.accentedness	0.0069	0.0011	6.314	<.001***
L1.comprehensibility	0.0088	0.0014	6.358	<.001***

**Table 4.** Result of model (1) for speakers with LOR = 0.

	Estimate	Std. Error	*Z*	*p*
L1.accentedness	-0.0018	0.0011	-1.533	.125
L1.comprehensibility	0.0142	0.0014	10.365	<.001***

To further examine the effect of LOR on L1 accentedness scores, we also fitted the following linear mixed effects (LME) model, using the *lme4* ( Bates et al., 2015) and *lmerTest* ( Kuznetsova et al., 2017) packages in R:

lmer(L1.accentedness∼LOR.months+(1|JP.rater),df.subset) (2)

where the dependent variable of L1 accentedness score was predicted by the fixed effect of the actual LOR (in months), with random intercepts for native Japanese raters. Note that only speakers with LOR > 0 were included in the model. The result is shown in Table 5. The significant positive estimate for the fixed effect confirms that speakers’ LOR in an L2-speaking environment predicts the degree of perceived L1 foreign accent, where an increase in LOR by 10 years (i.e., 120 months) is estimated to increase the L1 accentedness score by 12.17 (0.1014 × 120) on a 100-point scale.

**Table 5.** Result of model (2) for speakers with LOR > 0.

	Estimate	Std. Error	*Z*	*p*
LOR.months	0.1014	0.0008	12.507	<.001***

## 3. Acoustic analysis

The above analysis of the rating data suggests that more proficient L2 speakers tend to be perceived as more foreign-accented and yet more comprehensible in their L1 speech, except that those who have never lived abroad do not seem to gain an L1 foreign accent as they become more proficient in the L2. It remains to be seen, however, based on what acoustic characteristics the L1 speech was judged to be foreign-accented and comprehensible. To probe into this issue, we examine in this section the speakers’ L1 Japanese vowel production in relation to their accentedness and comprehensibility scores. We also refer to the same speakers’ L2 English vowel production and their nativelikeness scores to complement the analysis (cf. Yazawa et al., 2023a).

### 3.1 General procedure

**3.1.1 Materials**

The audio recordings of Tasks 6_01 and 6_02 in the J-AESOP corpus (see Section 2.2) were examined. The speech samples were annotated in Praat TextGrid format ( Boersma & Weenink, 2024), first by automatic forced alignment tools (HTK ( Young et al., 2006) for Task 6_01 and Julius ( Lee & Kawahara, 2019) for Task 6_02) and then manually modified by trained phoneticians in the J-AESOP team. Annotators also marked segment-level phonological events such as vowel devoicing (e.g., /kitakaze/ ‘North Wind’ ➔ [ki̥takaze]) and lengthening (e.g., /taijou/ ‘Sun’ ➔ [taijoː]) in Japanese, as well as word-level speech events such as substitution (e.g., misreading *cloak* as *coat* ), repetition (e.g., *Then the Sun … Then the Sun shone out warmly*), and insertion (e.g., *wrapped around in a warm cloak*) by assigning ‘tags’ to the relevant words (underlined above).

**3.1.2 Data retrieval and acoustic measurement**

Based on the annotations, a total of 34963 Japanese vowels (/i/ = 6504, /e/ = 3598, /a/ = 15713, /o/ = 6504, /u/ = 2444) produced by the 154 L1 Japanese speakers were retrieved for acoustic analysis. Their production of 10775 English monophthongal vowels (/i/ = 1591, /ɪ/ = 3196, /ɛ/ = 911, /æ/ = 1451, /ʌ/ = 1094, /ɑ/ = 1095, /u/ = 893, /ʊ/ = 544) was also retrieved. Devoiced and lengthened vowels, as well as vowels in tagged words, were excluded.

For each vowel interval, the mean F1 and F2 frequencies were measured using Praat. The built-in Burg algorithm was used for formant estimation, with the formant ceiling setting at 5000 Hz for male speakers and 5500 Hz for female speakers. The obtained F1 and F2 values were then Z-transformed per speaker ( Lobanov, 1971), which effectively eliminates spectral variations caused by physiological differences while preserving phonological and cross-linguistic contrasts ( Adank et al., 2004). The normalization was performed across Tasks 6_01 and 6_02 so that the formants could be directly compared across L1 Japanese and L2 English speech.

### 3.2 Acoustic correlates of accentedness

We begin our analysis by examining the potential acoustic correlates of perceived L1 foreign accent. Given the finding in Section 2 that L1 foreign accented was typical of L2 speakers with overseas experience, here we focus on the 55 speakers with LOR > 0. We first hypothesized that the formant values of L1 vowels in foreign-accented speech are dislocated from those of non- or less-accented speech, presumably due to the assimilation or dissimilation of L1 vowels to acquired L2 vowels. To test this, we used the following LME model:

lmer(L1.accentedness∼vowel:F1.norm+vowel:F2.norm+(1|JP.rater),df.subset) (3)

where the dependent variable of L1 accentedness score was predicted by the interaction between vowel type (/i, e, a, o, u/) and normalized formant values (F1 and F2), with random intercepts for native Japanese raters. The results are shown in Table 6, where all fixed effects turn out to be statistically significant. For example, the significant negative estimates for the interactions between the vowel /i/ and the two types of formant values indicate that lower F1 and F2 (i.e., raised and more back articulation) predict a higher L1 accentedness score.

**Table 6.** Result of model (3) for speakers with LOR > 0.

	Estimate	Std. Error	*t*	*p*
/i/:F1.norm	-0.8330	0.1110	-7.502	<.001***
/i/:F2.norm	-0.2822	0.0879	-3.210	.001**
/e/:F1.norm	-0.8796	0.2413	-3.646	<.001***
/e/:F2.norm	0.6577	0.1650	3.987	<.001***
/a/:F1.norm	0.5553	0.0852	6.517	<.001***
/a/:F2.norm	0.4195	0.1298	3.232	.001**
/o/:F1.norm	0.9986	0.1586	6.295	<.001***
/o/:F2.norm	-0.5908	0.0959	-6.163	<.001***
/u/:F1.norm	-0.4485	0.1589	-2.822	.005**
/u/:F2.norm	-0.5397	0.2380	-2.268	.023*

**Figure 4.**
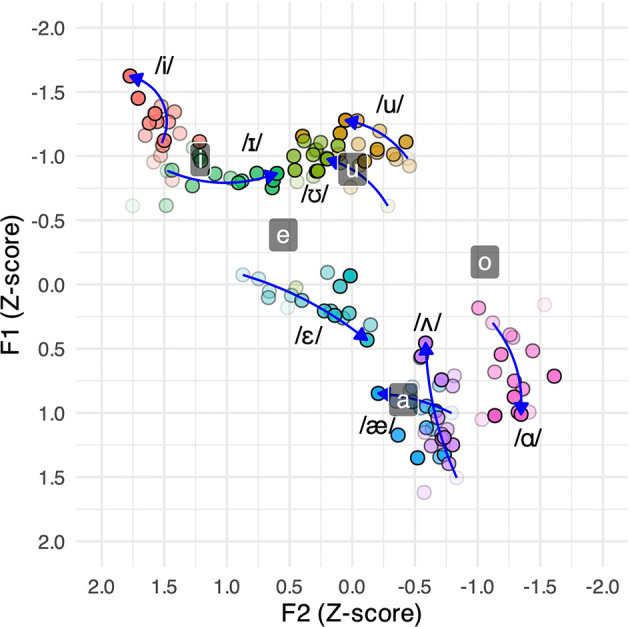
Japanese and English vowel formants of speakers with LOR > 0 according to L2 nativelikeness scores.

To complement the above results, we also show in Figure 4 the production of L2 English vowels by the same 55 speakers as a function of their L2 nativelikeness scores (cf. Yazawa et al. (2023a)). Each circle shows the mean Z-normalized formant values of a 0.50 score range (i.e., 1.25-1.75, 1.75-2.25, etc., as in the bins of Figure 1), with darker shades representing higher scores. The arrows point from lowest through intermediate to highest score ranges based on these means, ignoring apparent outliers. The mean formant values of L1 Japanese vowels are also shown alongside as gray boxes (averaged across the L2 score ranges because the by-range differences in F1 and F2 values were too subtle to plot). Since speakers with higher L2 nativelikeness scores were perceived as having a stronger L1 foreign accent for the current sample, a comparison of Table 6 and Figure 4 can be useful to examine how acquired L2 vowels might affect L1 vowel production.

The comparison shows that only some of the results can be straightforwardly explained by L1-L2 segmental assimilation or dissimilation. For example, the results for L1 /e/ in Table 6, where lower F1 and higher F2 values predict a higher L1 accentedness score, can be interpreted as a case of dissimilation from the adjacent L2 vowel /ɛ/, which shows the opposite pattern as a function of L2 nativelikeness score in Figure 4. That is, as speakers become more proficient in the L2 (and thus more accented in the L1), their L1 /e/ production is raised and fronted while that of L2 /ɛ/ is lowered and backed, resulting in an increased distance between the two categories. In contrast, the results for L1 /o/ in Table 6, where higher F1 and lower F2 values predict a higher L1 accentedness score, can be interpreted as a case of assimilation to L2 /ɑ/, which shows the same pattern according to L2 nativelikeness scores in Figure 4. That is, as speakers become more proficient in the L2 (and thus more accented in the L1), the L1 and L2 vowels move to the same direction (similar to what Turner (2023) called “tandem drift”). The results for the other three L1 vowels, however, were more mixed. As for /i/, while the negative estimate for F1 (i.e., raising) in Table 6 may be attributed to its assimilation to L2 tense /i/ (see Figure 4), the negative estimate for F2 (i.e., backing) would contrarily indicate dissimilation from this very L2 category. The same goes for L1 /u/, where the negative F1 estimate (i.e., raising) would indicate assimilation to L2 /u/ or /ʊ/, whereas the negative F2 estimate (i.e., backing) would indicate dissimilation from these two categories. L1 /a/ is a similar case, where the positive F1 estimate (i.e., lowering) would indicate dissimilation from L2 /æ/ or /ʌ/, while the positive F2 estimate (i.e., fronting) would indicate assimilation to these categories. The supposed assimilation-dissimilation patterns are, therefore, inconsistent at best.

Another explanation becomes possible when we shift our focus from individual L1-L2 category contrasts to the whole vowel space. Considering the suggested phonetic changes in Table 6—raising and backing of /i/, raising and fronting of /e/, lowering and fronting of /a/, lowering and backing of /o/, and raising and backing of /u/—one can envision a clockwise chain shift. Judging from Figure 4, such movements of L1 vowels seem to be helpful for maintaining their distance from the developing L2 categories.

### 3.3 Acoustic correlates of comprehensibility

We now move on to examine the potential acoustic correlates of perceived L1 comprehensibility. Here we investigate all 154 speakers, since more proficient L2 speakers were found to be more comprehensible in their L1 regardless of overseas experience in Section 2. The first acoustic parameter to be investigated is the ‘compactness’ of vowel categories in the F1-F2 acoustic space. Kartushina and Frauenfelder (2014) found that individuals with more compact L1 vowel categories tend to show more accurate L2 vowel production, presumably because the existing L1 categories overlap less with the target L2 vowels. SLM-r ( Flege & Bohn, 2021) provides a theoretical explanation of this finding through its “category precision” hypothesis, which posits that individuals with relatively precise L1 categories are better able to discern phonetic differences between an L2 sound and the closest L1 sound, thereby increasing the likelihood of new L2 category formation. If this is also true for the current sample, we can hypothesize that higher L1 comprehensibility in more proficient L2 learners is also due to their more compact or precise production of L1 vowel categories.

Following Kartushina and Frauenfelder (2014), we first calculated the compactness score (CS) per speaker and vowel type, using the following formula:

$$\mathrm{CS}=\sigma_{F_{1_{\mathrm{norm}}}}\sigma_{F_{1_{\mathrm{norm}}}}\pi$$ (4)

where $\sigma_{F_{1_{\mathrm{norm}}}}$ and $\sigma_{F_{2_{\mathrm{norm}}}}$ are the standard deviations of Z-normalized F1 and F2 values. The score thus reflects the area of the normalized vowel ellipse per speaker and vowel type. While Kartushina and Frauenfelder (2014) used raw formant values because they had only female speakers, we used Z-normalized values because our data contain both male and female speakers. Note that, somewhat counterintuitively, a larger CS represents a larger vowel ellipse and therefore a less compact category.

**Figure 5.**
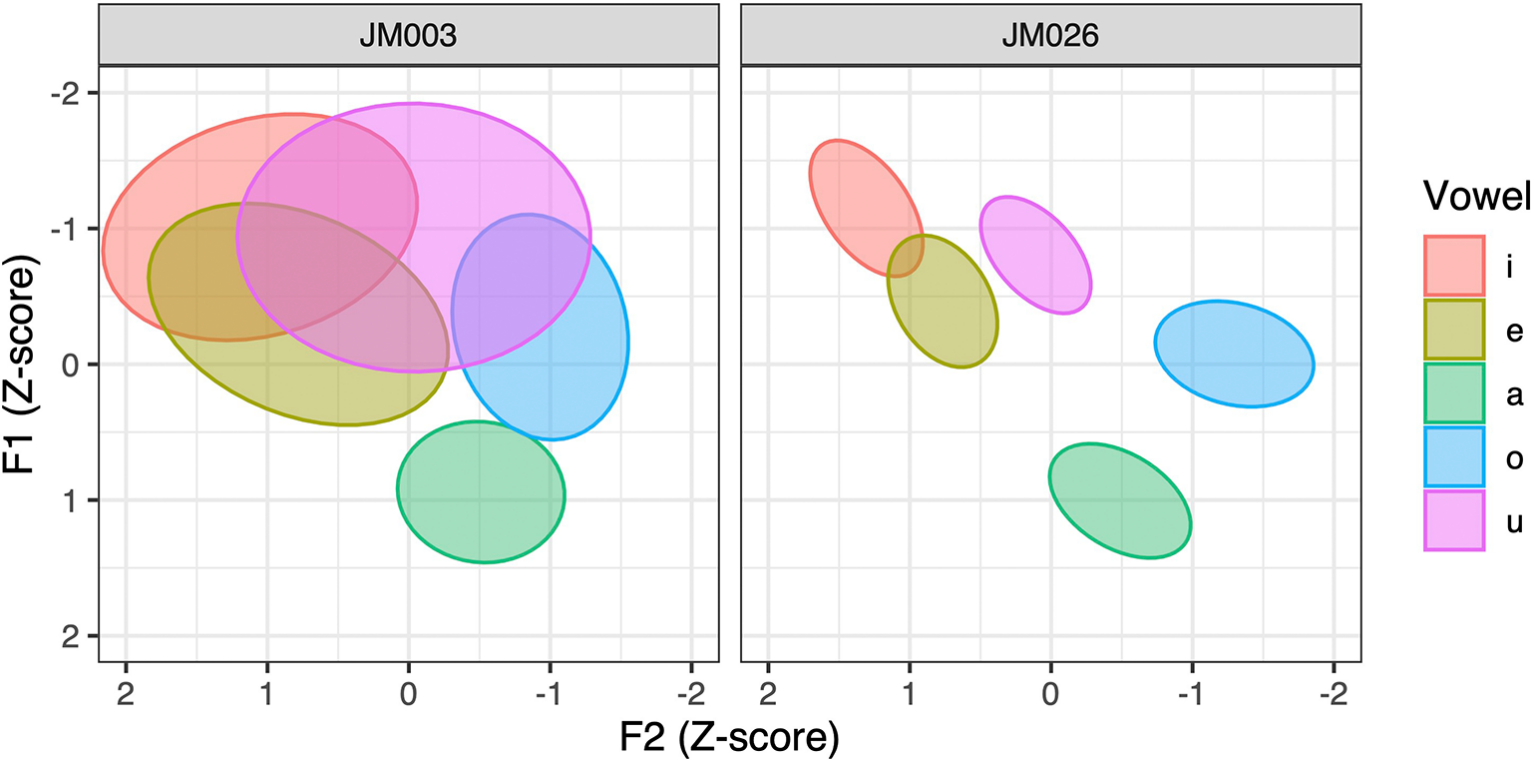
Vowel ellipses of two male speakers with less (left) and more (right) compact articulation.

The calculated five CSs were then added to obtain the global compactness score (CS _G_) per speaker:

$${\mathrm{CS}}_{G}=\sum_{i=1}^{n}{\mathrm{CS}}_{i}$$ (5)

where a larger CS _G_ would indicate generally less compact vowel categories. This is illustrated in Figure 5, which shows the vowel ellipses of two male speakers with relatively large and small CS _G_ values (6.71 for JM003 and 1.87 for JM026), respectively. It can be seen that the ellipses, which reflect one standard deviation of normalized F1 and F2 values, are less compact and more overlapping for the former speaker than for the latter.

Another acoustic parameter we will investigate here is formant dispersion, since compact vowel categories can still overlap with one another if they are not sufficiently dispersed and non-compact vowel categories can still be clearly distinguishable from each other if they are sufficiently dispersed. Thus, we can derive an alternative hypothesis that higher L1 comprehensibility in more proficient L2 learners is due to their more dispersed L1 vowel production.

**Figure 6.**
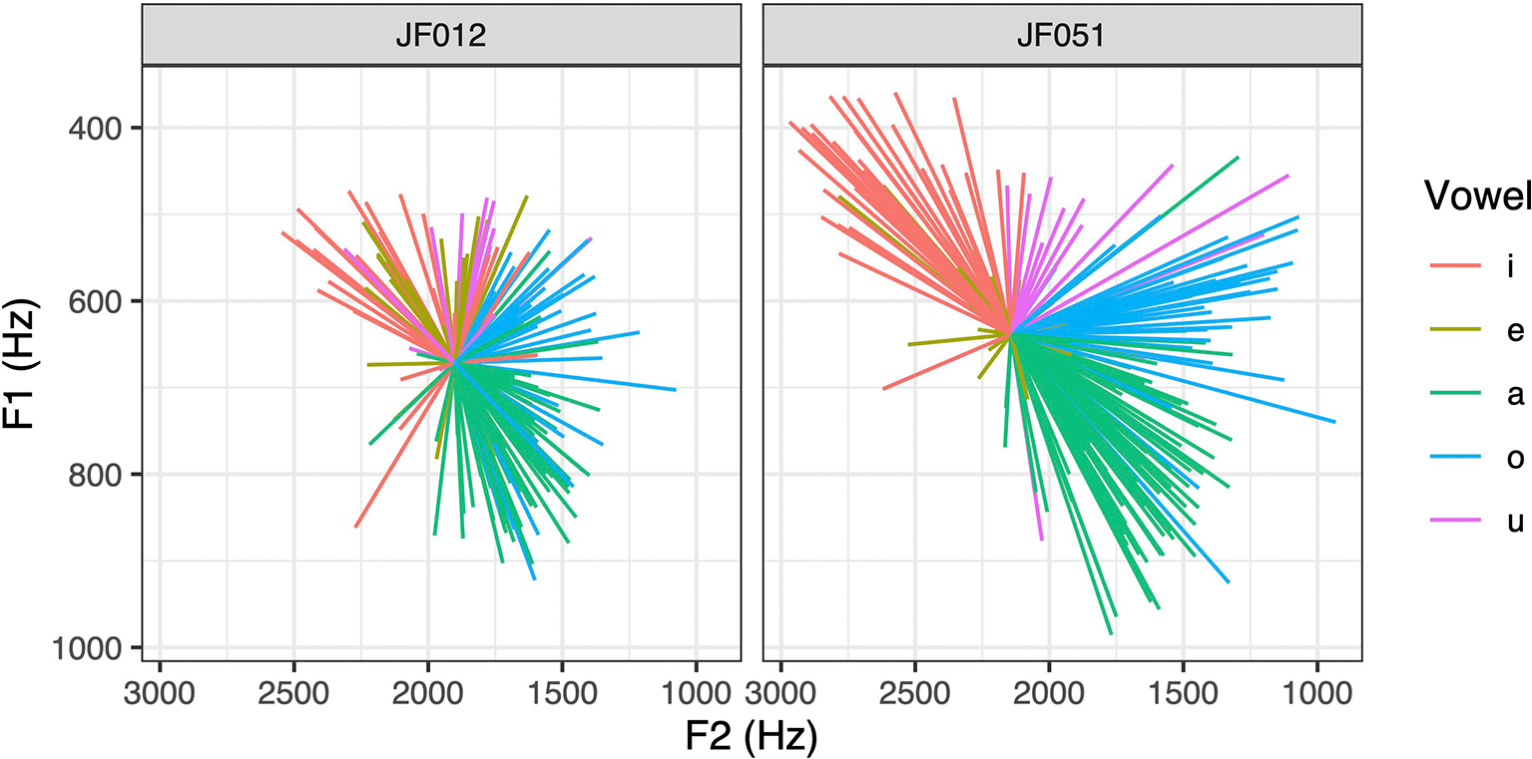
Vowel vectors of two female speakers with less (left) and more (right) dispersed articulation.

While there are several metrics for measuring formant dispersion, we used the vowel formant dispersion (VFD) metric of Karlsson and van Doorn (2012). To calculate the VFD, we first need to define the center of the F1-F2 vowel space for each speaker. The coordinate of the midpoint along the vertical (F1) axis is calculated according to:

$$F_{1_{m}}=\frac{1}{n}\sum_{i=1}^{n}F_{1_{i}}$$ (6)

for all vowels, and thus the midpoint is simply the mean of all F1 values. On the other hand, the position of the center along the horizontal (F2) axis is defined as a weighted midpoint:

$$F_{2_{\mathrm{wm}}}=\frac{1}{n}\sum_{i=1}^{n}F_{2_{i}}\left\{V\left(F_{2},F_{1}\right):F_{1}<F_{1_{m}}\right\}$$ (7)

and is therefore based only on vowels whose *F*_1_ value is lower than the *F*_1_ midpoint (i.e., more raised than the average vowel height). According to Karlsson and van Doorn (2012), this strategy makes the weighted center robust to missing productions of a suitable low front vowel, as in Japanese. Once the center of the *F*_1_- *F*_2_ vowel space is established, each vowel is converted into a vector starting from that point. Each vowel vector is defined to have a length according to:

$${\mathrm{VFD}}_{i}=\sqrt{{\left(F_{1_{i}}-F_{1_{m}}\right)}^{2}+{\left(F_{2_{i}}-F_{2_{\mathrm{wm}}}\right)}^{2}}$$ (8)

where the vector length, called VFD, is expected to increase for expanded vowel spaces and decrease for spaces with reduced articulation. This is illustrated in Figure 6, which shows vowel vectors of two female speakers with relatively small and large mean VFD values (268.98 for JF012 and 493.02 for JF051), respectively. Note that the values are in Hz because the effect of dispersion would be eliminated if they were Z-normalized.

Now that the acoustic parameters are defined, the question is whether individual differences in vowel compactness or dispersion predict the degree of perceived L1 comprehensibility. To test this, we used the following LME model:

lmer(L1.comprehensibility∼global.CS+mean.VFD+(1|JP.rater),df.all) (9)

where the fixed effects of CS _G_ and mean VFD, both of which are specific to each speaker, predicted their L1 comprehensibility score, with random intercepts for native Japanese raters. Although mean VFD tended to be larger for female speakers (because the metric is based on raw formant values), speakers’ gender was not included because it did not improve the model fit according to likelihood ratio tests. The result of the model is shown in Table 7. While both fixed effects were statistically significant, the positive estimate for CS _G_ contradicts our first hypothesis because a larger CS _G_ (i.e., less compact articulation) is associated with a higher L1 comprehensibility score. In contrast, the positive estimate for mean VFD suggests that more dispersed articulation predicts a higher L1 comprehensibility score, which is consistent with our second hypothesis.

**Table 7.** Result of model (9) for all speakers.

	Estimate	Std. Error	*t*	*p*
global.CS	0.5011	0.2091	2.397	.017*
mean. VFD	0.0287	0.0040	7.169	<.001***

To fully support the second hypothesis, however, we must also demonstrate that individuals with more dispersed L1 vowel production are more proficient in the L2. To test this, we fitted the following CLMM:

clmm(L2.nativelikeness∼global.CS+mean.VFD+(1|EN.rater),df.all) (10)

where the fixed effects of CS _G_ and mean VFD predicted the L2 nativelikeness score, with random intercepts for native American English raters. While only mean VFD would be relevant to the hypothesis testing, CS _G_ was also included in the model to test whether the result of Kartushina and Frauenfelder (2014) can be replicated with the current data. The result of the model is shown in Table 8, where neither effect reached statistical significance. The non-significant effect of CS _G_ is perhaps best illustrated in Figure 5, as the nativelikeness scores for the two speakers happen to be nearly equal (4.17 for JM003 and 4.25 for JM026). Therefore, our prediction that individuals with less overlapping L1 categories (due to compactness, dispersion, or both) would achieve a higher level of L2 proficiency based on the “category precision” hypothesis of SLM-r ( Flege & Bohn, 2021) was not supported. To sum up this section, higher comprehensibility in L1 speech is associated with more dispersed vowel articulation, but the degree of L1 vowel dispersion does not adequately predict L2 proficiency.

**Table 8.** Result of model (10) for all speakers.

	Estimate	Std. Error	*t*	*p*
global.CS	0.0163	0.0720	0.226	.821
mean. VFD	0.0014	0.0014	1.023	.307

## 4. General discussion

### 4.1 Overall summary

The primary purpose of the current study was to investigate the potential effects of L2 learning on perceived accentedness and comprehensibility of L1 speech. To this end, we examined 154 L1 Japanese learners of L2 English in the J-AESOP corpus with varied L2 learning experience and proficiency levels. The rating results revealed that more proficient L2 speakers tended to be perceived as more foreign-accented in the L1, but only if they had lived in an English-speaking country; no such relationship was found for those who had never lived abroad. Subsequent acoustic analyses of vowel production suggested that the degree of perceived foreign accent could not be straightforwardly attributed to either assimilation or dissimilation of individual L1 and L2 categories but rather to a system-wide phonetic change. In contrast, more proficient L2 speakers were consistently perceived as more comprehensible in the L1, regardless of the presence or absence of prior overseas experience. Acoustic analyses suggested that the degree of perceived comprehensibility was associated with the dispersion, rather than compactness, of vowel production, although neither dispersion nor compactness in L1 vowel production predicted L2 proficiency levels.

### 4.2 Implications

**4.2.1 Accentedness**

The rating results for accentedness suggest that L2 learners do not necessarily gain an L1 foreign accent as they become proficient in the L2. Rather, strong L1 accentedness seems to be associated with the experience of living in an L2-speaking (or non-L1-speaking) environment for an extended period of time. Therefore, to answer our first research question, a perceptible L1 foreign accent is likely due to L1 disuse rather than L2 interference. It may thus be misleading to refer to this phenomenon as “backward transfer” because the acquired L2 system is not directly affecting the existing L1 system; the term “attrition” would be more appropriate in this sense. It should be noted, however, that the degree of perceived L1 foreign accent for the current sample was less profound than that of the migrant populations in the previous studies ( de Leeuw et al., 2010; Kornder, 2022). This may be because our speakers maintained their L1 oral skills through everyday conversations with their parents and siblings while abroad and/or partially reversed L1 attrition after re-immersion into the L1-speaking environment back in Japan. While the LOR information in the corpus does not reflect these possibilities, the effects of relative L1 exposure while abroad and the length of L1 re-immersion in Japanese-English bilingual returnees is extensively discussed in Laméris et al. (2024).

Our acoustic analysis of vowel production also indicated an intriguing pattern of potential L1 phonetic change in proficient L2 speakers with overseas experience: clockwise chain shift in the F1-F2 space. While no previous study has reported such a pattern, our finding would not contradict previous reports of system-wide phonetic change due to drift or attrition ( Guion, 2003; Mayr et al., 2012; Turner, 2023). Thus, to answer our second research question, perceived L1 accent seems to reflect a system-wide change in all sound categories, rather than individual assimilation or dissimilation of adjacent categories between the two languages. Caution is warranted, however, because the previous studies observed a systematic lowering or raising of all vowel categories, whereas in the current study the direction of change varied from vowel to vowel (similar to Kornder (2022)). Nevertheless, the motivation for these diverging patterns may be the same, i.e., to maintain a perceptual distinction between the L1 and L2 sounds, as proposed by Guion (2003).

**4.2.2 Comprehensibility**

A novel finding of this study was that a higher level of L2 proficiency consistently predicted a higher level of L1 comprehensibility (consistent with our previous pilot study ( Yazawa et al., 2023b)). This was somewhat surprising given the negative correlation between L1 accentedness and comprehensibility (cf. Figure 2), but perhaps not entirely unexpected since a strong foreign accent does not necessarily reduce perceived comprehensibility for L2 speech ( Munro & Derwing, 1995). Thus, to answer our final research question, the influence of L2 learning on perceived L1 comprehensibility can be positive. Logically speaking, there are two possible explanations for the current finding: (i) learners whose L1 speech is comprehensible tend to acquire a high level of L2 proficiency, and (ii) as learners become more proficient in the L2, their L1 speech becomes more comprehensible. The first possibility was theoretically predicted by the “category precision” hypothesis of SLM-r ( Flege & Bohn, 2021), as outlined in Section 3.3. However, the acoustic analyses did not support this prediction, since neither compactness nor dispersion of L1 vowel articulation was related to L2 nativelikeness scores (in contrast to Kartushina and Frauenfelder (2014) who found a link between L1 vowel compactness and L2 proficiency). It is then worth noting that the VFD metric did predict the perceived degree of L1 comprehensibility, because if this viable acoustic predictor of L1 comprehensibility does not predict L2 proficiency, then (i) is not very feasible. The alternative possibility is (ii), which, although no current model of L2 speech acquisition offers an explicit explanation for it, seems plausible in nature. If L2 learning involves an effort to clearly articulate speech sounds to make oneself better understood, then learners may extend this skill to their L1 production, thus increasing its comprehensibility (even though the resulting speech may differ from monolingual norms). If such an effect turns out to be true, then this L2-related ‘amelioration’ or ‘enrichment’ of L1 speech would be an important advantage of bilingualism, especially in the current era where comprehensibility is considered more important than nativelikeness.

### 4.3 Concluding remarks

In this study, we conducted a large-scale cross-sectional investigation of L1 phonetic change in native Japanese speakers with various L2 English learning experience and proficiency levels using the J-AESOP corpus. The overall results seem to suggest two main conclusions. First, perceived L1 foreign accent likely results from L1 disuse rather than L2 interference, where a learner’s L1 pronunciation is shifted from monolingual norms at a system-wide rather than category-specific level. Second, L2 learning can have a positive influence on perceived L1 comprehensibility, rather than individuals whose L1 speech is more comprehensible being better equipped for L2 learning. However, the current investigation is limited in that the corpus data do not inform us of how the learners’ pronunciation has actually changed over time; we are, after all, comparing different speakers from different linguistic backgrounds. What seems necessary to confirm and extend the current research, then, is a longitudinal study of intra-speaker acoustic-phonetic change and its communicative impact, similar to previous studies (e.g., Kornder (2022)) but with a larger number of speakers. To this end, a longitudinal bilingual speech corpus, which is far less common than cross-sectional ones such as J-AESOP, is expected to be useful. While building such a corpus would be labor-intensive and time-consuming, we believe that the benefits outweigh the effort. A preliminary project towards this goal has been initiated, and we look forward to sharing what it brings about in the future.

## Ethics and consent

This study was reviewed and approved by the Research Ethics Committee of the Institutes of Humanities and Social Sciences, University of Tsukuba on May 17, 2022 (approval number 2022-3) and was conducted in accordance with the ethical standards of the Helsinki Declaration. Participants signed a written consent form prior to participation.

## Data Availability

Zenodo: J-AESOP Rating Data (6_01 & 6_02). https://doi.org/10.5281/zenodo.10633636 ( Konishi & Yazawa, 2024). This project contains the following underlying data.
• Rating6_01.csv • Rating6_02.csv The demographic information and audio samples of the speakers in the J-AESOP corpus cannot be publicly shared because open posting of data on a repository was not included in the consent form at the time of data collection.
