# Supplementary material for: More foreign-accented but more comprehensible: Attrition and amelioration of L1 speech in proficient L2 learners
Source: F1000Res. 2024 Aug 1;13:867. [Version 1] doi: 10.12688/f1000research.148193.1 (PMC11413555; doi:10.12688/f1000research.148193.1)
Supplement: Peer review history

# Reviewer report: Reviewer response for version 1

**DOI:** 10.5256/f1000research.162474.r320620

**Published:** 2024-09-19

**Authors:** Enrique Cámara-Arenas

**Affiliations:** 1 University of Valladolid, Valladolid, Spain

A relevant and well-designed piece. It is concerned with determining elements that influence that backward acquisition of non-native features in L1 pronunciation possibly relatable to the attainment of L2 proficiency. Improving over existing literature on similar topics is the consideration of L1 comprehensibility.

 The conclusions are properly extracted and immediately relevant to the field. There is, as expected, an increase of L1 accentedness that corresponds to L2 proficiency, but seems causally related to a lack of use of the L1 for an extended period of time. The influence of the L2 over the L1 does not respond to a segment by segment projection of L2 units onto similar L1 units, but rather to a systemic economy apparently guided by the need to differentiate and separate L1 and L2 phonological repertoires.

 Interestingly, L1 comprehensibility appears to be enhanced with L2 proficiency.

 There are aspects of this research which I cannot fully judge, especially those concerned with the rigor of statistic treatment and procedure.

 The piece is, however, relevant, well written, convincing in its argumentation, and satisfactory in its conclusions.

Is the work clearly and accurately presented and does it cite the current literature?

Yes

If applicable, is the statistical analysis and its interpretation appropriate?

I cannot comment. A qualified statistician is required.

Are all the source data underlying the results available to ensure full reproducibility?

No

Is the study design appropriate and is the work technically sound?

Yes

Are the conclusions drawn adequately supported by the results?

Yes

Are sufficient details of methods and analysis provided to allow replication by others?

Yes

Reviewer Expertise:

Teaching of L2 English pronunciation

I confirm that I have read this submission and believe that I have an appropriate level of expertise to confirm that it is of an acceptable scientific standard.

# Reviewer report: Reviewer response for version 1

**DOI:** 10.5256/f1000research.162474.r311386

**Published:** 2024-09-04

**Authors:** Takako Kawasaki

**Affiliations:** 1 Hosei University, Chiyoda Ward, Japan

Yazawa et al. (2024) investigate three key questions in the field of second language acquisition and first language attrition. First, they explore whether a foreign accent in the first language (L1) results from improvements in the second language (L2) or simply from reduced use of L1. Second, they examine whether the L1 foreign accent reflects changes in specific sound categories or a broader shift in the entire sound system. Lastly, they investigate how L2 learning impacts L1 comprehensibility.

 To address these questions, Yazawa et al. analyzed a corpus containing speech samples from native Japanese speakers in both their native language (Japanese) and their second language (English). Japanese native speakers evaluated the foreign accent and comprehensibility of the L1 Japanese speech, while native English speakers assessed the nativeness of the L2 English speech. Additionally, the researchers conducted acoustic analyses of the Japanese and English vowels produced by each speaker.

 The findings are intriguing. The study concludes that the L1 foreign accent is more likely to arise from reduced L1 use rather than L2 interference, suggesting that the foreign accent in L1 is more a result of language attrition due to decreased L1 usage, rather than an effect of improved L2 skills. Furthermore, they found that the accent changes in L1 pronunciation represent a shift at the system-wide level, rather than changes in specific sound categories.

 The paper uses a methodical approach to these complex questions, employing appropriate methodologies. It provides a thorough review of previous research and offers insightful results and discussion. The authors also acknowledge the limitations of their study, particularly those related to the characteristics of the corpus, and suggest directions for future research.

 This study contributes valuable insights to our understanding of how L2 learning can affect L1 pronunciation and comprehensibility. It challenges some common assumptions in the field, particularly with its finding that L1 foreign accent is more likely due to L1 attrition rather than L2 interference.

Is the work clearly and accurately presented and does it cite the current literature?

Yes

If applicable, is the statistical analysis and its interpretation appropriate?

I cannot comment. A qualified statistician is required.

Are all the source data underlying the results available to ensure full reproducibility?

Yes

Is the study design appropriate and is the work technically sound?

Yes

Are the conclusions drawn adequately supported by the results?

Yes

Are sufficient details of methods and analysis provided to allow replication by others?

Yes

Reviewer Expertise:

Phonology, SLA, Phonetics

I confirm that I have read this submission and believe that I have an appropriate level of expertise to confirm that it is of an acceptable scientific standard.
